# The role of metamemory and personality in episodic memory performance in older adults

**DOI:** 10.1007/s40520-023-02341-x

**Published:** 2023-01-28

**Authors:** Graziana Lenti, Elena Carbone, Enrico Sella, Kristin E. Flegal, Erika Borella

**Affiliations:** 1 Department of General Psychology, University of Padova, Padua, Italy; 2 Institute of Neuroscience and Psychology, University of Glasgow, Glasgow, UK

**Keywords:** Episodic memory, Metamemory, Personality, Memory strategy, Aging

## Abstract

This study newly investigated the joint contribution of metamemory and personality (traits and facets) in explaining episodic memory (EM) performance in typically aging older adults. Forty-eight participants (age range: 64–75 years) completed a self-paced word list (SPWL) recall task, a metamemory questionnaire assessing perceived control and potential improvement (PCPI) and self-efficacy and satisfaction (SESA) regarding one’s mental abilities (e.g., memory), and the Big-Five Questionnaire. Based on the SPWL encoding strategies reported, participants were then classified as effective (*N* = 20) or ineffective (*N* = 28) memory strategy users. Hierarchical regression analyses showed that a better SPWL performance was predicted by higher levels of PCPI, Scrupulousness and Dominance personality facets. Effective memory strategy users, then, showed higher SPWL performance and Dominance (Energy facet) than ineffective ones. These findings suggest that both specific metamemory processes and personality facets predict better EM performance in older adults. Moreover, personality dispositions relating to Dominance seem to characterize individuals adopting effective memory strategies to support EM performance. These results represent first evidence of the role of both metamemory and personality—facets—in explaining older adults’ EM performance, which should thus be considered when assessing or training EM in old age.

**Supplementary Information:**

The online version contains supplementary material available at 10.1007/s40520-023-02341-x.

## Introduction

Episodic memory (EM) is among those memory systems sensitive to age-related changes with aging [1]. Older adults’ impairments in EM tasks, such as recalling word lists (WL), is, in fact, well documented.

Beside classical explanations for EM impairments in aging, including a decrease in the mental resources available for self-initiated deep encoding processes, or an associative processes deficit [1], there is also the impact of metacognitive processes involved in memory functioning, or metamemory [2, 3]. Metamemory refers to one’s knowledge of, beliefs about and awareness of development, functioning and capacities of one’s memory [4]. In particular, metamemory processes encompass various related components, such as memory self-efficacy and controllability–perceived control–(estimated capability of memory, and efficient memory use and management), monitoring skills (awareness and control processes of memory), feeling of satisfaction with memory (satisfaction with one’s memory), and metamemory knowledge (how memory functions as well as strategies’ knowledge, and their use) [4, 5]. Changes in metamemory components due to aging might affect older adults’ effort to control their “learning through action” [6] and hence their EM performance. Perceived control (PC) over one’s cognitive and memory functioning, and memory self-efficacy (MSE) were, for instance, shown to be positively associated with performance in EM tasks in adulthood and older age [7–9]. Moreover, though addressed by only one study, older individuals who performed better in WL recall tasks were found to more likely report being satisfied with their memory [10].

More recent research has also focused on the relationship between personality traits (as conceived by the theoretical framework of the Big-Five model [11]) and older adults’ cognitive functioning [12]. In particular, some personality traits—more—related to the adoption of healthy lifestyles, engagement in cognitively stimulating activities, and response to stress were shown to have a “protective role” against age-related changes in cognitive abilities, including EM [13]. Higher levels of Openness (O) and Conscientiousness (C), and lower levels of Neuroticism (N), for instance, were shown to account for a better WL recall performance in older adults [14–16]. Evidence is mixed, however, regarding the role of other personality traits, such as Extraversion (E) and Agreeableness (A). E was positively associated with WL recall performance in some studies [15], but negatively in others [14]. Though usually found scarcely related to older adults’ cognition and EM [12, 14], A was found associated with older adults’ WL recall performance in some studies—either positively [16, 17] or negatively [18].

Apart from major traits, some few studies have examined also the association between narrower facets of personality in older age, which are not simply interchangeable with the broad traits they are designed to reflect [19, 20], and EM. Chapman et al. [21] found a better older adults’ WL recall performance related to higher levels of Intellectual Interest (O facet), Goal-Striving and Dependability (C facets). Their study also showed positive associations between EM and Activity (E facet) and Orderliness (C facet). Aiken-Morgan et al. [17] found also Positive Emotion (E facet) and Deliberation (C facet) negatively, while Straightforwardness (A facet) positively associated with EM performance in a verbal learning task. In contrast, Graham and Lachman [22] reported no associations between personality facets and EM performance in young and older adults.

In sum, although some—few—researches have been carried out on metamemory and EM [7–10], and on personality and cognitive (memory) performance [12]; surprisingly, no studies have jointly considered metamemory and personality in relation to EM performance in older adults. Moreover, concerning metamemory, the literature has likely focused on specific isolated components (i.e., PC, MSE) without considering others*–*satisfaction with one’s memory*–*that could also be relevant for EM functioning. Therefore, these complementary metacognitive components are worth investigating in concert. Regarding personality, considering narrower personality facets, which allow for depiction of different and more nuanced associations with cognition, would help further capture and reveal associations between personality and EM performance [12, 23, 24], an aspect that few researchers have examined.

The aim of this study–a pilot one–was therefore to examine whether, and to what extent, both metamemory processes (in particular PC, MSE, and satisfaction with memory) because of their influence in EM performance, and personality (traits and facets) play a role in explaining WL recall performance in a sample of typically aging older adults.

We also investigated whether metamemory processes and personality could be related to individual differences in older adults’ spontaneous use of more or less effective memory strategies to support their EM.

In line with the literature, we expected WL recall performance to be associated with PC and MSE [7–9]. In line with Troyer and Rich [10], we also expected a positive association between older adults’ satisfaction with their mental abilities, such as memory, and their WL recall performance. As for personality traits, we expected WL recall performance to be associated positively with O and C, and negatively with N [14–16]. Given the previous contrasting results [14, 15, 17, 18], we further explored the relationships between E, A, and EM performance. We also hypothesized an association between older adults’ WL recall performance and narrower facets of personality characterizing O, C, E, and A [17, 22], an aspect on which studies’ results are still unclear.

As for memory strategy use, being better EM performance positively related to the adoption of deep encoding processes, we expected effective memory strategies users to have a better EM performance [25, 26]. We then explored whether effective and ineffective memory strategy users differed in the metamemory processes and personality traits and facets considered here. According to previous evidence on EM performance in young and older adults [27, 28], we could predict that effective memory strategies users would display higher adaptive metamemory processes like PC, and personality dispositions (O in particular) than ineffective strategy ones.

Since EM performance is influenced by background characteristics such as education level and vocabulary [15, 25], these aspects were treated as control variables in all the analyses run.

## Method

### Participants

Forty-eight community dwellers Italian older adults (age range: 64–75 years; 52% females) volunteered for the study. All participants were recruited in southern Italy between May 2019 and January 2020 at social clubs, or by word of mouth.

The inclusion criteria were the following: (i) good physical and mental health, assessed with a semi-structured interview [29]; (ii) scores ≥ 27 on the Mini-Mental State Examination (MMSE) [30]; (iii) scores ≤ 5 on the Geriatric Depression Scale (GDS) [31]; (iv) scores within the norm for the Wechsler Vocabulary test [32]. All participants had a typical aging process (see Table 1).

**Table 1:** Mean (M) and standard deviations (SD) of the demographic characteristics and screening measures

	M	SD
Age (years)	68.37	3.46
Education (years)	12.81	3.10
Vocabulary	44.23	9.60
Mini-Mental State Examination	27.53	0.44
Geriatric Depression Scale	1.63	1.58

The study was approved by the local ethics committee for the psychological research (protocol number 3277). The experimental procedure complied with the principles of the Declaration of Helsinki (1964 and later amendments).

## Materials

*Big-Five Questionnaire-60* (BFQ-60) [33]. It consists of 60 items. Participants were asked to indicate to what extent they felt described by each statement on a 5-point Likert scale from 1 (very false for me) to 5 (very true for me). They evaluate the five major personality traits and their ten related facets: Dynamism (activity and enthusiasm) and Dominance (assertiveness and self-confidence) for Energy (E); Scrupulousness (orderliness and precision) and Perseverance (commitments and persistence) for C; Emotion Control (absence of anxiety or depression) and Impulse Control (ability to control irritation and anger) for Emotional Stability (ES); Cooperativeness (altruism and empathy) and Politeness (kindness and trust) for Agreeableness (A); Openness to Culture (intellectual curiosity, and interest in being informed); and Openness to Experience (openness to novelty, interest in different people, and lifestyles) for O. The final scores for the personality traits and facets were obtained by averaging the corresponding items’ scores.

*Metamemory questionnaire*. It is an ad hoc questionnaire designed for the present study, consisting of 24 items borrowed -and/or adapted- from well-known questionnaires (see Supplemental Materials–Part 1) to examine metamemory processes of PC, MSE, and satisfaction about one’s cognitive abilities, such as memory. Participants were asked to indicate their agreement with each statement on a 7-point Likert scale from 1 (strongly disagree) to 7 (completely agree).

A factor analysis was conducted to explore the questionnaire’s structure using principal component factor extraction with Varimax rotation and applying Kaiser’s eigenvalue greater than one rule to derive common underlying factors. The Kaiser–Meyer–Olkin measure of sampling adequacy was 0.67, and Bartlett’s test of sphericity was significant (*χ*^*2*^_(276)_ = 622.76; *p* < 0.001). The Kaiser’s eigenvalue greater than one rule was applied, and six factors accounting for 68.8% of the variance in the data were identified with eigenvalues > 1.0. The subsequent analysis of the Scree plot revealed two factors with eigenvalues above the elbow of the graph [34], and factor loadings higher than 0.50 were used to interpret these two factors (see Supplemental Materials–Part 1 for further details). The first factor was labeled “self-efficacy and satisfaction” (SESA; Cronbach’s alpha = 0.84), and the second factor was labeled “perceived control and potential improvement” of one’s memory abilities (PCPI; Cronbach’s alpha = 0.90). The dependent variables were thus the sum of the answers for the items in each component, with higher scores corresponding to higher SESA and PCPI.

*Self-paced word list* (SPWL). The list consists of 20 words presented auditorily with a self-paced procedure. Participants pressed a button on a computer mouse to hear the next word in a series. Then, they had to recall as many words as possible from the list. The number of correctly recalled words was considered.

*Strategy use questionnaire*. After the SPWL task, participants were asked whether they had used particular procedures to learn the WL. The use of strategies freely reported by participants was classified as follows [25, 35]: 0 = *None* (i.e., unsuccessful attempts to use a strategy, or no reference to any strategy use), 1 = *Marginal* (repetition, acronyms); 2 = *Optimal* (semantic associations, mental imagery, or a mix of marginal and optimal strategies).

### Procedure

Each participant attended a single 90-min individual session. They read and signed the informed consent form, and then completed the measures in the following order: a semi-structured interview about physical and mental health status; the MMSE; the Vocabulary; the SPWL; the Strategy use questionnaire; the BFQ-60; the Metamemory questionnaire; and the GDS.

### Statistical analyses

The analyses were conducted using IBM SPSS (Version 28), and the probability level was set at *p* < 0.05.

Spearman’s correlations were run between all the measures of interest.

Then, hierarchical regressions were conducted to elucidate the contribution of metamemory and personality in explaining older adults’ SPWL performance. Participants’ sociodemographic characteristics [age, vocabulary (crystallized intelligence), and education] were controlled for given their influence on older adults’ EM performance, and added in Step 1. The other predictors of interest were added in the subsequent steps order: metamemory (PCPI, SESA) was entered in Step 2, and broader individual personality dispositions—traits (in a first set of analyses) or facets (in a second)—in Step 3.

Finally, independent samples *T *tests were run to explore whether participants reporting no or ineffective strategies (No or Marginal), or effective ones (Optimal), differed in terms of metamemory and personality.

## Results

Descriptive statistics of the measures of interest are shown in Table S2 (Supplementary Materials–Part 2).

### Correlations

No significant correlations emerged between age and the other variables (Supplementary Materials–Part 3). Education showed medium positive correlations with vocabulary (*r* = 0.48; *p* < 0.01) and Openness to Culture (*r* = 0.40; *p* < 0.01), and a weak negative correlation with Perseverance (*r* = − 0.33; *p* < 0.05); vocabulary revealed medium positive correlations with the A trait (*r* = 0.41; *p* < 0.01) and its Cooperativeness (*r* = 0.33; *p* < 0.05) and Politeness facets (*r* = 0.39; *p* < 0.01), and a negative correlation with Perseverance (*r* = − 0.33; *p* < 0.05). Negative correlation emerged between SESA, A (*r* = − 0.32; *p* < 0.05), and Cooperativeness (*r* = − 0.32; *p* < 0.01), while no significant correlations emerged between PCPI and the other variables.

### Regression analyses

In the hierarchical regression analysis with metamemory–PCPI, SESA–and personality traits as predictors, none of the predictors explained performance in the SPWL task (Supplementary Materials–Part 4).

When metamemory–PCPI, SESA–and personality facets were entered as predictors (see Table 2), all the predictors explained 49% of the variance in SPWL performance, and the final model was significant. Step 1 and Step 2 did not account for any significant portions of variance in EM performance. The largest and most significant portion of the variance in SPWL performance was explained by personality facets in Step 3 (37%). PCPI, and the Dominance and Scrupulousness facets of personality emerged as significant positive predictors in the final model^1^.

### Strategy use

The sample was divided into two groups: “ineffective -or no- strategy users” (*n* = 28) and “optimal strategy users” (*n* = 20). The results (see Table 3; see Supplementary Materials–Part 5) showed that the two groups did not differ in terms of background variables, such as age, education, and vocabulary scores. The optimal strategy users significantly recalled more words than ineffective strategy users, *t*_(1,46)_ = − 4.86; *p* < 0.001, *d* = − 1.40. 2Optimal strategy users reported also higher Dominance levels than ineffective strategy ones, *t*_(1,46)_ = − 2.08; *p* < 0.05, d = − 0.60.

**Table 2:** Hierarchical regression analysis with demographic variables (age, education, and vocabulary) (step 1), metamemory factors (step 2), and personality facets (step 3) as predictors of performance in the SPWL recall task

	SPWL (words recalled)						
	Model 1	Model 2	Model 3				
	*β*	*β*	*β*	B	95% CI (−)	95% CI ( +)	VIF
Age	− 0.229	− 0.229	− 0.207	− 0.150	− 0.361	− 0.062	1.309
Education	0.034	0.048	− 0.225	− 0.181	− 0.493	0.130	2.275
Vocabulary	0.187	0.162	0.216	0.056	− 0.040	0.152	2.063
PCPI		0.193	0.307*	0.087*	0.003	0.172	1.334
SESA		0.042	0.052	0.018	− 0.090	0.126	1.533
Dynamism			− 0.272	− 1.062	− 2.607	0.482	2.374
Dominance			0.494**	2.414**	0.824	4.004	1.612
Scrupulousness			0.546**	2.631**	0.940	4.322	1.874
Perseverance			− 0.096	− 0.409	− 1.845	1.026	1.711
Emotion Control			0.143	− 0.454	− 0.827	1.736	2.472
Impulse Control			− 0.133	− 0.492	− 2.130	1.145	2.998
Cooperativeness			− 0.087	− 0.398	− 2.023	1.227	1.925
Politeness			0.172	0.664	− 1.056	2.383	3.015
Openness to Culture			0.299	1.334	− 0.224	2.892	1.855
Openness to Experience			− 0.193	− 0.816	− 2.277	0.646	1.829
_*R*_^2^	0.085	0.124	0.493* 0.369*	*F*_(*15,32*)_ = 2.078, *p* = 0.04			
Δ*R*^2^		0.039					

*R*^2^, *ΔR*^2^, and standardized β concern each step, B and 95% CI concern the last step (model 3). *SPWL* self-paced word list, *PCPI* perceived control and potential improvement, *SESA* self-efficacy and satisfaction

*p <0 .05; **p < 0.01

## Discussion

This study newly investigated the association between typically aging older adults’ EM–WL–recall performance, and their metamemory processes (particularly PC, MSE, and satisfaction with their memory) and the Big-Five personality traits, and their facets. We also examined whether any of these aspects accounted for individual differences in older adults’ spontaneous use of more or less effective memory strategies to support EM performance (Table 3).

**Table 3:** Differences between ineffective and optimal strategy use groups in the variables of interest

	Ineffective strategy users (*n* = 28)		Optimal strategy users (*n* = 20)		*T* tests			Mann–Whitney tests	
	*M*	*SD*	*M*	*SD*	*t*_*(1,46)*_	*p*	*d*	*Z*	*p*
Age*	68.29	3.56	68.50	3.41	− 0.21	0.42	− 0.06	− 0.29	0.76
Education*	12.25	2.98	13.60	3.17	− 1.51	0.07	− 0.43	− 1.63	0.10
Vocabulary	43.25	8.74	45.60	1.78	− 0.83	0.20	-0.24		
SPWL (number of words recalled)	4.18	1.95	7.10	2.20	− 4.86	<0 .001	− 1.40		
PCPI*	50.36	10.48	51.85	5.77	− 0.58	0.28	− 0.17	− 0.12	0.90
SESA	36.82	7.52	36.20	7.27	0.29	0.39	0.08		
ENERGY*	3.05	0.41	3.14	0.42	− 0.69	0.25	− 0.20	− 0.67	0.50
Dynamism	3.75	0.69	3.62	0.63	0.71	0.24	0.20		
Dominance	2.36	0.50	2.66	0.49	− 2.08	0.02	0-0.60		
CONSCIENTIOUSNESS	3.87	0.54	3.82	0.39	− 0.351	0.36	0.10		
Scrupulousness	3.88	0.55	3.96	0.47	− 0.49	0.32	− 0.14		
Perseverance	3.85	0.62	3.68	0.53	− 0.98	0.17	0.28		
EMOTIONAL STABILITY	3.29	0.60	3.28	0.76	− 0.101	0.46	0.03		
Emotion Control	3.30	0.73	3.32	0.88	− 0.08	0.47	− 0.002		
Impulse Control	3.29	0.66	3.23	0.72	− 0.08	0.39	0.08		
AGREEABLENESS	3.65	0.55	3.72	0.51	− 0.45	0.33	− 0.13		
Cooperativeness*	3.69	0.60	3.84	0.47	− 0.91	0.19	− 0.26	− 0.76	0.44
Politeness	3.60	0.61	3.59	0.71	0.02	0.49	0.01		
OPENNESS	3.75	0.52	3.72	0.45	0.22	0.41	0.06		
Openness to Culture*	3.73	0.62	3.89	0.45	− 1.01	0.16	− 0.29	− 0.63	0.52
Openness to Experience*	3.78	0.56	3.55	0.62	1.34	0.09	0.38	− 1.56	0.11

*Variables that, according to the results of the Shapiro–Wilk test, do not show normal distributions. *PCPI* perceived control and potential improvement, *SESA* self-efficacy and satisfaction, *SPWL* self-paced word list

Results from the regression analyses, in line with the correlations, showed that neither metamemory processes nor personality traits were associated with EM performance. This lack of any EM performance–metamemory processes’ associations, though in contrast with previous findings [8, 27], aligns with other reports [7, 9]. Such contrasting results could be due to the variables considered and controlled in the analyses, which may have prevented the role of the metamemory processes considered from emerging [7, 9]. As discussed below, the expected role of metamemory in WL recall intriguingly emerged when personality facets were examined.

For personality, an unexpected lack of association, inconsistent with reports of relationships between personality traits and EM in older adults [14, 15], was found. However, this might be due to the EM tasks used, which appear to vary in terms of complexity between our own and previous studies. A self-paced encoding time was used here, whose successful management demands monitoring and control processes [36], unlike the tasks based on “constrained” encoding times adopted in other studies [16]. Our task also involved the recall of a longer WL (20 words) than in other studies using lists of 16, 15, or 10 words [14, 15, 18], and the latter coincided with proportionally better performance (with at least 50% of words recalled correctly, as opposed to 30% in our case). These are only speculations, however, that merits to be further investigated when examining the relationship between personality traits and older adults’ EM performance, by manipulating WL task’s complexity (e.g., using WL of different lengths and presenting them at different rates).

The age range chosen for this study may also help to explain our results. Researchers who found significant associations between personality traits and EM examined samples with wider age ranges (49–90 years [17], 50–107 years [14], and 60–78 years [16]) than the range investigated here (64–75 years). Although personality traits are assumed to remain fairly stable individual characteristics [37], they have been known to exhibit different lifespan trajectories [38]. The narrower age range considered here might therefore have prevented us from detecting any associations between personality traits and EM performance. At the same time, our narrower age range and a more homogeneous education level in our sample may also explain why age, education, and vocabulary did not predict EM performance, as reported elsewhere [14, 27].

On the other hand, newly exploring the role of personality facets, related but not simply interchangeable with the broad traits they reflect, better captured the contribution not only of specific dimensions of personality, but also of some metamemory processes in explaining a large part of the variance (37%) in SPWL performance. In line with the previous reports of personality facets characterizing C and E being positively associated with EM in older age [17, 22], we found that Scrupulousness (C facet) and Dominance (E facet) contributed to explaining WL recall performance. When personality facets were entered in the regression model, PCPI also emerged to be associated with performance in the SPWL task. These findings suggest that orderliness and precision, as well as assertiveness, and a tendency to compete and work hard to excel—alongside a greater perceived control of one’s resources and potential to improve one’s performance—might predispose older adults to be more focused in an EM task. This would in turn enable them to concentrate their resources on managing the task successfully, thereby favoring their subsequent performance. These characteristics seem to be particularly useful to older adults when they are needed to complete (as in our case) a relatively demanding task (20 words to be remembered) that necessitates good self-management, control, and monitoring processing (self-paced). Our results therefore confirm that older adults’ perceived control and belief in their potential to improve their mental abilities play a part in influencing EM performance [7].

As suggested by previous studies [14, 16], our findings underscore the role of certain personality dispositions in “protecting” against age-related changes in EM, and promoting a good performance in WL recall tasks. They also seem to confirm that personality facets can have a specific variance unrelated to their broader dimensions [19, 20], which can shed light on the associations between personality and cognition [12, 23, 24]. Our results thus underscore the need to examine not only broader but also narrower dimensions of personality more systematically in an effort to better capture the contribution of personality to older adults’ EM performance, when a self-paced procedure is used at least.

The role of the SESA–the other metamemory component assessed–did not emerge in our analyses. This could be due to the MSE characteristics identified by our questionnaire. Previous meta-analyses [8, 9] have established that there is a significant but quite small association between MSE and EM performance in young and older adults. Such an association becomes weaker when global MSE (general beliefs about memory abilities) is considered, as done here, rather than concurrent/specific MSE (beliefs specific to a given task and the context of its realization). Moreover, alongside MSE, SESA also concerns satisfaction with one’s mental abilities, such as memory, whose (presumably positive) link with older adults’ EM performance is still unclear and understudied [10]. Further research is therefore needed to clarify the part played by such metamemory aspects in EM, also in relation to personality, and the different (global or concurrent) ways in which MSE could be operationalized.

Examining metamemory processes and personality dispositions comparing older adults who used more or less effective memory strategies supported the relationship between personality and EM performance. Participants who reported optimal strategies not only recalled more words than those using ineffective or no strategies, but exhibited also higher Dominance levels. These findings suggest that personality characteristics related to dominance (e.g., willingness to compete and show one’s skills) have a role in older adults’ EM, prompting individual differences in their effective, spontaneous use of memory strategies to enhance performance in the WL recall task. In contrast with the only other study examining this issue [28], we did not find a higher O associated with the use of effective memory strategies in older adults. The two studies used a very different test procedure, however. Talpain and Soubelet [28] administered a cued-recall task presenting a list of 20 pairs of words to be memorized during a fixed encoding time. It would be worth investigating whether the interplay between personality and strategy use varies as a function of the EM task’s features (and the consequently different demands and processes involved).

Contrary to expectations and other reports [27], optimal strategy users did not have higher PCPI than ineffective strategy users. These results are consistent, however, with one previous study by Hertzog et al. [35], which found that personal control beliefs were not significantly related to deep encoding strategy use in older adults. Future studies should therefore try to clarify the relationship between metamemory and older adults’ spontaneous use of more or less effective memory strategies. Differences in the characteristics of the samples, such as age range and education level, and in the way PC is operationalized and strategy use is categorized [25, 27, 35] could account for the different results reported to date.

Some limitations of this study, that should be considered as a pilot one, must be acknowledged. First, the sample size was small. A larger sample size with larger age range is needed in future studies to more comprehensively capture age-related changes in EM performance and the potential influence of individual characteristics in terms of metamemory processes and personality dispositions. Then, we did not examine the role of other, potentially relevant metacognitive aspects, such as beliefs and attributions regarding memory functioning [25] and mindset [39], which could influence older adults’ cognitive functioning. It would also be worth exploring, as stated, whether the pattern of predictors of EM performance changes when the WL tasks have different features (e.g., constrained vs. self-paced encoding times, number of words in the list), or when other, more ecological EM tasks are used (e.g., story recall).

To conclude, certain metamemory processes (perceived control and beliefs regarding potential for improvement) and personality dispositions (facets of C and E) were found to be associated with EM, in terms of WL recall performance at least. Our findings also newly suggest that specific personality facets related to assertiveness and self-confidence explain individual differences in terms of older adults’ spontaneous use of more or less effective memory strategies. From an applied-research and clinical perspective, our results suggest the importance of systematically examining metamemory processes and personality dispositions when assessing EM performance. Further, personality characteristics and metamemory processes might also help define the profile of individuals who could benefit most from EM training to design more effective and tailored interventions to support such an age-sensitive memory system so fundamental in everyday life.

## Supplementary Information

Below is the link to the electronic supplementary material.

Supplementary file1 (DOCX 1567 KB)

## Data Availability

The data that support the conclusions of this article will be made available by the corresponding authors upon reasonable request.

## Footnotes

1 Considering all variables included in the model (see Table 2), a power analysis showed that an estimated sample of 51 people was needed to obtain a sufficient power of .80, an effect size of .35, and a p<.05 (using the R software’s *pwr* library). We gathered 60 participants initially, but only 48 were eligible according to the inclusion criteria, slightly under the minimum required sample size.

2 This category was created by merging 8 participants who had not used any kind of strategy and 20 participants who used ineffective strategies (marginal strategy users). These two groups did not differ in their demographic characteristics or any of the measures of interest (ps >.10; see Table S5). Excluding the 8 “no-strategy users” from the analyses did not change the results.
