# Supplementary material for: The role of metamemory and personality in episodic memory performance in older adults
Source: Aging Clin Exp Res. 2023 Jan 28;35(3):669–76. doi: 10.1007/s40520-023-02341-x (PMC10014676; doi:10.1007/s40520-023-02341-x)
Supplement: Supplementary file 1 — Supplementary file1 (DOCX 1567 KB) [file 40520_2023_2341_MOESM1_ESM.docx]

**Supplementary Materials**

**The role of metamemory and personality in episodic memory performance in older adults.**

*Summary of the contents*

PART 1 – Details of the Metamemory Questionnaire

PART 2 – Descriptive statistics of the sample’s demographics and measure of interest

PART 3 – Correlations between the measure of interest

PART 4 – Personality traits as predictor of episodic memory performance

PART 5 – Descriptive statistics for no strategy and ineffective strategy users

**PART 1 – DETAILS OF METAMEMORY QUESTIONNAIRE**

**Design of the Metamemory Questionnaire and results from exploratory factor analysis**

The Metamemory Questionnaire is an ad hoc questionnaire comprising 24 items borrowed -and/or adapted- from well-known metamemory questionnaires (Memory Controllability Inventory –MCI –, Lachman et al., 1995; Memory in Adulthood Questionnaire –MIA–, Dixon & Hultsch, 1983; Multifactorial Memory Questionnaire –MMQ–, Troyer & Rich, 2002) to examine metamemory processes.

In particular, for Memory Self-Efficacy (MSE) 3 items (e.g., “I can remember the things I need to”) were taken from the Present Ability subscale of the MCI, and another two were created ad hoc, taking inspiration always from the Present Ability subscale of the (e.g., “I can use my mental resources, such as memory, attention, in a flexible way; that is, I can find alternative ways of dealing with a given situation”). Perceived Control (PC) was assessed using 3 items from the MCI- Effort Utility subscale (e.g., “If I work at it, I can improve my memory”) and 3 from the MCI- Potential Improvement subscale (e.g., “I can find ways to improve my memory”), and adapting 7 items from the Locus subscale of the MIA (e.g., “I know if I keep using my mental abilities, such as memory, attention, I will never lose them”). Satisfaction with one’s cognitive abilities, such as memory, was assessed by adapting 6 items of the Contentment subscale of the MMQ (e.g., “I am satisfied with the functioning of my mental abilities, such as memory, attention”).

Figure S1 shows the scree plot of eigenvalues obtained from principal component analysis run on the 24 items of the questionnaire to explore its factor structure. In Table S1 factor loadings are presented.

**Figure S1. *
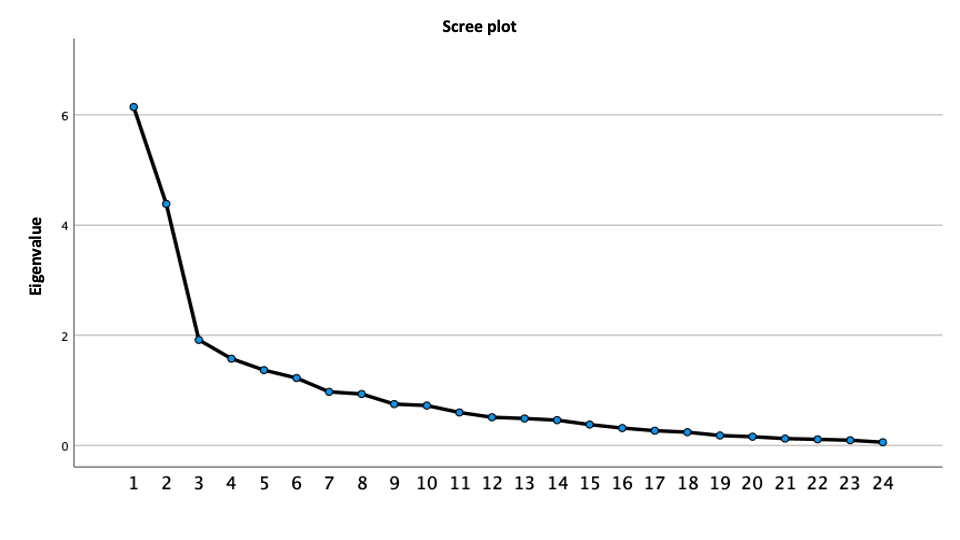
****Scree plot of eigenvalues obtained from principal component analysis on the 24 items of the metamemory questionnaire.*

One item from the Present Ability subscale of the MCI and one ad-hoc item assessing memory self-efficacy, along with five items from the Contentment subscale of the MMQ assessing satisfaction with one’s cognitive abilities such as memory, loaded onto one factor, which explained 25.60% of the variance (eigenvalue = 6.14). This factor was thus labeled “self-efficacy and satisfaction (SESA)” towards one’s own memory abilities.

Three items from the Effort Utility subscale and two items from the Potential Improvement subscale of the MCI, along with five items from the Locus subscale of the MIA, assessing perceived control over one’s memory ability, loaded onto the other second factor. One item was omitted to increase the reliability of the PCPI rating (see Table S1). This second factor, explaining 18.28% of the variance (eigenvalue = 4.39) was thus labeled “perceived control and potential improvement (PCPI)” of one’s own memory abilities.

The internal consistency of the final 16-item questionnaire was excellent for both SESA (Cronbach’s alpha = .84) and PCPI (Cronbach’s alpha = .90) components.

**Table S1.** *Results of factor analysis (Varimax rotation method) on the 24 items of the metamemory questionnaire.*

| **24 items** | **Factor 1 (PCPI)** | **Factor 2 (SESA)** |
| --- | --- | --- |
| 1 | .049 | .323 |
| 2 | .073 | **.654** |
| 3 | **.508** | .133 |
| 4R* | -.158 | **.753** |
| 5 | -.027 | .432 |
| 6 | **.556** | .040 |
| 7R* | .431 | .131 |
| 8R* | .294 | .166 |
| 9R* | .169 | .362 |
| 10R* | -.006 | **.597** |
| 11R* | .105 | **.806** |
| 12 | .053 | .489 |
| 13 | **.837** | -.240 |
| 14 | .175 | **.615** |
| 15 | **.839** | -.053 |
| 16 | **.654** | -.157 |
| 17 | .045 | .493 |
| 18R* | -.002 | **.723** |
| 19 | **.784** | -.090 |
| 20 | **.841** | .127 |
| 21 | **.752** | .146 |
| 22 | **.772** | .242 |
| 23 | .094 | **.730** |
| 24 | **.806** | .136 |

Note. Factor loadings higher than .50 (in bold) were used to interpret the factors.

*R stands for Reverse. The item 3 was deleted to increase reliability of PCPI to .90.

**PART 2 - DESCRIPTIVE STATISTICS OF THE SAMPLE’S DEMOGRAPHICS AND MEASURE OF INTEREST**

**Table S2.** Mean (M) and standard deviations (SD) of the measures of interest.

|  | *M* | *SD* |
| --- | --- | --- |
| Episodic memory |  |  |
| SPWL (number of words recalled) | 5.40 | 2.50 |
| Metamemory |  |  |
| PCPI | 50.98 | 8.78 |
| SESA | 36.56 | 7.34 |
| Personality |  |  |
| ENERGY | 3.09 | 0.41 |
| Dynamism | 3.69 | 0.64 |
| Dominance | 2.48 | 0.51 |
| CONSCIENTIOUSNESS | 3.85 | 0.48 |
| Scrupulousness | 3.91 | 0.52 |
| Perseverance | 3.78 | 0.58 |
| EMOTIONAL STABILITY | 3.29 | 0.66 |
| Emotion control | 3.31 | 0.79 |
| Impulse control | 3.27 | 0.68 |
| AGREEABLENESS | 3.68 | 0.53 |
| Cooperativeness | 3.76 | 0.55 |
| Politeness | 3.59 | 0.65 |
| OPENNESS | 3.74 | 0.49 |
| Openness to culture | 3.80 | 0.56 |
| Openness to experience | 3.68 | 0.59 |

Note. SESA: self-efficacy and satisfaction; SPWL: self-paced word list.

**PART 3 - CORRELATIONS BETWEEN THE MEASURE OF INTEREST**

**Table S3.** Spearman’s correlations between the measures of interest.

|  | 1 | 2 | 3 | 4 | 5 | 6 | 7 | 8 | 9 | 10 | 11 | 12 | 13 | 14 | 15 | 16 | 17 | 18 | 19 | 20 |
| --- | --- | --- | --- | --- | --- | --- | --- | --- | --- | --- | --- | --- | --- | --- | --- | --- | --- | --- | --- | --- |
| 1. Age | 1 |  |  |  |  |  |  |  |  |  |  |  |  |  |  |  |  |  |  |  |
| 2. Education | -.10 | 1 |  |  |  |  |  |  |  |  |  |  |  |  |  |  |  |  |  |  |
| 3. Vocabulary | .14 | .51** | 1 |  |  |  |  |  |  |  |  |  |  |  |  |  |  |  |  |  |
| 4. PCPI | -.03 | .01 | .09 | 1 |  |  |  |  |  |  |  |  |  |  |  |  |  |  |  |  |
| 5. SESA | .24 | -.05 | .02 | .05 | 1 |  |  |  |  |  |  |  |  |  |  |  |  |  |  |  |
| 6. Energy | -.15 | .06 | .08 | -.05 | .09 | 1 |  |  |  |  |  |  |  |  |  |  |  |  |  |  |
| 7. Dynamism | -.13 | -.09 | .06 | .16 | -.03 | .79** | 1 |  |  |  |  |  |  |  |  |  |  |  |  |  |
| 8. Dominance | -.09 | .22 | .05 | -.27 | .18 | .64** | .02 | 1 |  |  |  |  |  |  |  |  |  |  |  |  |
| 9. Conscientiousness | .08 | -.26 | -.18 | .10 | -.06 | -.01 | .21 | -.28 | 1 |  |  |  |  |  |  |  |  |  |  |  |
| 10. Scrupulousness | .09 | -.17 | -.03 | .09 | -.15 | .08 | .32* | -.27 | .85** | 1 |  |  |  |  |  |  |  |  |  |  |
| 11. Perseverance | .04 | -.28 | -.27 | .09 | .04 | -.09 | .06 | -.22 | .88** | .49** | 1 |  |  |  |  |  |  |  |  |  |
| 12. Emotional stability | .19 | -.12 | -.03 | -.03 | .09 | -.15 | -.03 | -.20 | .34* | .32* | .27 | 1 |  |  |  |  |  |  |  |  |
| 13. Emotion control | .10 | -.02 | -.12 | -.15 | .12 | -.14 | -.10 | -.09 | .28 | .20 | .28 | .92** | 1 |  |  |  |  |  |  |  |
| 14. Impulse control | .26 | -.22 | .07 | .12 | .04 | -.13 | .05 | -.28 | .34* | .40** | .20 | .89** | .63** | 1 |  |  |  |  |  |  |
| 15. Agreeableness | -.02 | .19 | .41** | .24 | -.37* | .17 | .49** | -.33* | .17 | .32* | .00 | .18 | .04 | .30* | 1 |  |  |  |  |  |
| 16. Cooperativeness | -.04 | .16 | .33* | .19 | -.38** | .05 | .27 | -.25 | .15 | .27 | .01 | .18 | .06 | .29* | .86** | 1 |  |  |  |  |
| 17. Politeness | .00 | .17 | .39** | .23 | -.28 | .23 | .57** | -.33* | .15 | .29* | -.01 | .13 | .02 | .24 | .90** | .56** | 1 |  |  |  |
| 18. Openness | -.11 | .17 | .21 | .10 | -.07 | .21 | .28 | .00 | .19 | .18 | .15 | .01 | .03 | -.02 | .34* | .34* | .26 | 1 |  |  |
| 19. Openness to culture | -.23 | .39** | .22 | .13 | -.14 | .11 | .11 | .04 | .05 | .07 | .02 | -.08 | -.02 | -.14 | .27 | .28 | .21 | .84** | 1 |  |
| 20. Openness to experience | .03 | -.09 | .14 | .03 | .01 | .25 | .36* | -.03 | .28 | .24 | .24 | .09 | .07 | .09 | .29* | .29* | .24 | .86** | .44** | 1 |
| 21. SPWL (words recalled) | -.21 | .15 | .17 | .22 | .00 | .19 | .03 | .28 | .19 | .29* | .04 | .02 | .02 | .01 | .05 | .04 | .06 | .18 | .31* | .00 |

Note. PCPI: perceived control and potential improvement; SESA: self-efficacy and satisfaction; SPWL: Self-paced word list.

p < .05 *; p < .01**

**PART 4 - PERSONALITY TRAITS AS PREDICTOR OF EPISODIC MEMORY PERFORMANCE**

**Table S4.** Hierarchical regression analysis with demographic variables (age, education and vocabulary) (step 1), metamemory factors (step 2) and personality traits (step 3) as predictors of performance in SPWL recall task.

|  | SPWL (words recalled) | | | | | | |
| --- | --- | --- | --- | --- | --- | --- | --- |
|  | Model 1 | Model 2 | Model 3 | | | | |
|  | β | β | β | B | 95% CI  (-) | 95% CI  (+) | VIF |
| Age | -.229 | -.229 | -.215 | -.155 | -.387 | .076 | 1.214 |
| Education | .034 | .048 | .099 | .080 | -.205 | .365 | 1.474 |
| Vocabulary | .187 | .162 | .265 | .069 | -.032 | .170 | 1.779 |
| PCPI |  | .193 | .234 | .067 | -.022 | .155 | 1.147 |
| SESA |  | .042 | -.067 | -.023 | -.142 | .097 | 1.452 |
| Energy |  |  | .199 | 1.204 | -.720 | 3.128 | 1.198 |
| Conscientiousness |  |  | .258 | 1.356 | -.418 | 3.130 | 1.348 |
| Emotion stability |  |  | .081 | .305 | -.952 | 1.563 | 1.305 |
| Agreeableness |  |  | -.266 | -1.260 | -3.190 | .671 | 1.967 |
| Openness |  |  | .047 | .239 | -1.425 | 1.902 | 1.252 |
| *R*^2^ | .085 | .124 | .238  .113 | *F* _(_*_10,37_*_)_ = 1.154, *p* = .35 | | |  |
| Δ*R*^2^ |  | .039 |  |  |  |  |  |

Note. R^2^, ΔR^2^ and standardized β concern each step, while B and 95% CI concern the last step (model 3). SPWL: self-paced word list; PCPI: perceived control and potential improvement; SESA: self-efficacy and satisfaction. *p < .05; **p < .01.

**PART 5 – DESCRIPTIVE STATISTICS FOR NO STRATEGY AND INEFFECTIVE STRATEGY USERS**

**Table S5.** Descriptive statistics of demographic characteristics and the measures of interest by no-stragey users and ineffective strategy users groups.

|  | No strategy users  (n = 8) | | Ineffective strategy users  (n = 20) | |
| --- | --- | --- | --- | --- |
|  | *M* | *SD* | *M* | *SD* |
| Age | 68.25 | 2.49 | 68.30 | 3.96 |
| Education | 13.38 | 3.89 | 11.80 | 2.51 |
| Vocabulary | 44.63 | 7.54 | 42.70 | 9.30 |
| Episodic memory |  |  |  |  |
| SPWL (number of words recalled) | 3.75 | 2.32 | 4.35 | 1.81 |
| Metamemory |  |  |  |  |
| PCPI | 46.88 | 10.49 | 51.75 | 10.41 |
| SESA | 36.13 | 9.54 | 37.10 | 6.82 |
| Personality |  |  |  |  |
| ENERGY | 3.07 | .45 | 3.05 | .41 |
| Dynamism | 3.67 | .69 | 3.78 | .65 |
| Dominance | 2.48 | .52 | 2.31 | .49 |
| CONSCIENTIOUSNESS | 3.80 | .49 | 3.89 | .56 |
| Scrupulousness | 3.83 | .54 | 3.90 | .57 |
| Perseverance | 3.77 | .50 | 3.88 | .67 |
| EMOTIONAL STABILITY | 3.13 | .70 | 3.36 | .55 |
| Emotion control | 3.21 | 1.01 | 3.33 | .61 |
| Impulse control | 3.04 | .77 | 3.39 | .60 |
| AGREEABLENESS | 3.69 | .60 | 3.63 | .54 |
| Cooperativeness | 3.81 | .59 | 3.65 | .61 |
| Politeness | 3.56 | .67 | 3.61 | .61 |
| OPENNESS | 3.83 | .54 | 3.72 | .53 |
| Openness to culture | 3.88 | .71 | 3.67 | .60 |
| Openness to experience | 3.79 | .49 | 3.78 | .60 |

Note. PCPI: perceived control and potential improvement; SESA: self-efficacy and satisfaction; SPWL: self-paced word list.

**References**

Lachman ME, Bandura M, Weaver SL, Elliott E (1995) Assessing memory control beliefs: The memory controllability inventory. Aging Neuropsychol Cogn; 2(1):67-84. doi: 10.1080/13825589508256589

Dixon RA, Hultsch DF (1983) Structure and development of metamemory in adulthood. J Gerontol; 38(6):682-688. doi: 10.1093/geronj/38.6.682

Troyer AK, Rich JB (2002) Psychometric properties of a new metamemory questionnaire for older adults.  J Gerontol B Psychol Sci Soc Sci; 57(1):P19-P27. doi: https://doi.org/10.1093/geronb/57.1.P19
